# Metabotyping of Docosahexaenoic Acid - Treated Alzheimer’s Disease Cell Model

**DOI:** 10.1371/journal.pone.0090123

**Published:** 2014-02-27

**Authors:** Priti Bahety, Yee Min Tan, Yanjun Hong, Luqi Zhang, Eric Chun Yong Chan, Pui-Lai Rachel Ee

**Affiliations:** Department of Pharmacy, National University of Singapore, Singapore, Republic of Singapore; Oregon Health & Science University, United States of America

## Abstract

**Background:**

Despite the significant amount of work being carried out to investigate the therapeutic potential of docosahexaenoic acid (DHA) in Alzheimer’s disease (AD), the mechanism by which DHA affects amyloid-β precursor protein (AβPP)-induced metabolic changes has not been studied.

**Objective:**

To elucidate the metabolic phenotypes (metabotypes) associated with DHA therapy via metabonomic profiling of an AD cell model using gas chromatography time-of-flight mass spectrometry (GC/TOFMS).

**Methods:**

The lysate and supernatant samples of CHO-wt and CHO-AβPP_695_ cells treated with DHA and vehicle control were collected and prepared for GC/TOFMS metabonomics profiling. The metabolic profiles were analyzed by multivariate data analysis techniques using SIMCA-P+ software.

**Results:**

Both principal component analysis and subsequent partial least squares discriminant analysis revealed distinct metabolites associated with the DHA-treated and control groups. A list of statistically significant marker metabolites that characterized the metabotypes associated with DHA treatment was further identified. Increased levels of succinic acid, citric acid, malic acid and glycine and decreased levels of zymosterol, cholestadiene and arachidonic acid correlated with DHA treatment effect. DHA levels were also found to be increased upon treatment.

**Conclusion:**

Our study shows that DHA plays a role in mitigating AβPP-induced impairment in energy metabolism and inflammation by acting on tricarboxylic acid cycle, cholesterol biosynthesis pathway and fatty acid metabolism. The perturbations of these metabolic pathways by DHA in CHO-wt and CHO-AβPP_695_ cells shed further mechanistic insights on its neuroprotective actions.

## Introduction

Alzheimer’s disease (AD) is an emerging public health concern for the global aging population. AD is an irreversible, chronic neurodegenerative disease, characterized predominantly by the presence of neuritic plaques of amyloid beta (Aβ) peptide and neurofibrillary tangles (NFTs) in the brain. The exact etiology of neurodegeneration is unknown but has been proposed to involve the interplay of mitochondrial dysfunction, inflammation, oxidative stress, excitotoxicity and NFT formation. To date, there is no disease-modifying treatment for AD. Recent evidence has suggested the potential advantages of polyunsaturated fatty acids (PUFAs) in AD, triggering questions on the contribution of diet in the management of this devastating disorder [1], [2]. PUFAs are bioactive molecules with diverse physiological functions ranging from its contribution in cell structure to signal transduction. Amongst the omega-3 (*n*−3) PUFAs, docosahexaenoic acid (DHA) has been found to be the most abundant in the mammalian brain, constituting about 8% of its dry weight [3]. It plays a key role in memory, neuroprotection and vision and displays inflammation resolving properties [4]. However, DHA levels have been found to be significantly decreased in serum and neuronal membranes of AD patients as compared to healthy controls [5], [6], suggesting a possible role of DHA in the intervention of AD. Based on the same hypothesis, lipidomics-based approaches have identified neuroprotectin D1, resolvins and maresin as the potent metabolites of DHA having anti-inflammatory and neuroprotective effects [4], [7]. It has also been demonstrated that DHA and these metabolites are efficacious in reducing Aβ plaque burden and protecting against learning impairment in animal models [8]–[11]. In view of the potential therapeutic benefits associated with DHA, there is an impetus to elucidate its role in preventing AD.

Impaired mitochondrial functions and hypometabolism of the neuronal cells have been characterized as neuropathological hallmarks of AD. Accumulation and deposition of amyloid precursor protein (AβPP) and its cleaved fragment, Aβ, have been implicated in the inhibition of various mitochondrial enzymatic activities and for perpetration of oxidative stress-mediated damages [12]–[15]. AβPP localizes within the mitochondrial membranes and causes disruption of mitochondrial homeostasis and functioning of the electron transport chain (ETC) [12], [16]–[18]. All these mitochondrial damages ultimately prevent the neuronal cells from functioning normally, leading to neuronal cell death. While the anti-inflammatory role of DHA has been investigated, its effect against AβPP-induced metabolic disturbances has not been studied. The aim of this study was to elucidate the mechanism of action of DHA in mitigating AβPP-induced metabolic perturbations by characterizing and comparing the metabotypes of DHA-treated and untreated wild type and AβPP-associated cells.

The quantitative measurement of metabolites and their perturbations offer insights into disease processes and pharmacological responses to therapeutic interventions. Gas chromatography time-of-flight mass spectrometry (GC/TOFMS) techniques have been well established and widely applied for the non-targeted profiling of endogenous metabolites [19]. Coupled to the chemical library databases and chemometric data analysis, GC/TOFMS-based metabonomics facilitates detection of the metabolic fluxes related to pharmacological interventions.

To address the aim of this study, a global metabonomics profiling of the medium and lysate of DHA- and vehicle-treated Chinese hamster ovary wild type (CHO-wt) cells and AβPP_695_-transfected CHO (CHO-AβPP_695_) cells was performed using the GC/TOFMS metabonomics. Our findings demonstrate for the first time that DHA plays a role in mitigating AβPP-induced impairment in energy metabolism and inflammation and shed preliminary mechanistic insights on its neuroprotective effects.

## Materials and Methods

### Materials

Dulbecco’s modified eagle’s medium (DMEM), succinic acid, glycine, citric acid, malic acid, mefenamic acid, silanization solution (5% dimethyl dichlorosilane in heptane) and sodium sulfate were purchased from Sigma-Aldrich (St Louis, MO, USA). Docosahexaenoic acid (DHA) and arachidonic acid were obtained from Cayman Chemical Co. (Ann Arbor, MI, USA). Zeocin was purchased from Invitrogen (Carlsbad, CA, USA). Fetal bovine serum (FBS) was obtained from Thermo Scientific Hyclone (Logan, UT, USA). *N*-Methyl-*N*-(trimethylsilyl)trifluoroacetamide (MSTFA) with 1% trimethylchlorosilane (TMCS) and 2% methoxamine hydrochloride in pyridine (MOX reagent) were purchased from Pierce (Rockford, IL, USA). Pyruvate dehydrogenase assay kit was purchased from Assay BioTech (Sunnyvale, CA, USA). Ultra-pure grade phosphate buffer saline (PBS) was obtained from 1^st^ Base Private Limited (Singapore). Dimethyl sulfoxide (DMSO), toluene and methanol were purchased from MP Biomedicals (Santa Ana, California, USA). Analytical grade reagents were used for all the work.

### Cell Culture

CHO-wt and CHO-AβPP_695_ cells were kindly provided by Associate Professor Gavin S. Dawe (Department of Pharmacology, Yong Loo Lin School of Medicine, National University of Singapore) [20] and cultured and maintained in DMEM medium supplemented with 10% FBS, 10 U/mL penicillin G, 100 µg/mL streptomycin and 0.4 mg/mL zeocin for CHO-AβPP_695_ cells, in humidified atmosphere at 37°C containing 5% CO_2._

### Western Blotting Analysis

Western blotting was used to examine the expression of AβPP gene in CHO-wt and CHO-AβPP_695_ cells. Both the cell types were seeded at a density of 1 × 10^5^ cells/2 mL of medium in 6 well plates. 24 h post-seeding the cells were harvested and lysed in lysis buffer containing 1% Triton with protease inhibitor (Roche, Mannheim, Germany). Protein concentration was determined by Bradford protein assay (Sigma Chemical Co., St Louis, MO, USA). 30 µg of protein lysates were then loaded in 10–15% polyacrylamide gel, prepared using 30% acrylamide-bisacrylamide (BioRad, Hercules, CA, USA). The separated proteins were subsequently blotted onto nitrocellulose membranes (Bio-Rad, Hercules, CA, USA) and blocked overnight at room temperature in tris-buffered saline containing 0.1% (*v*/*v*) Tween 20 and 5% (*w*/*v*) fat-free dry milk, followed by incubation with the anti-APP primary antibody (Merck KGaA, Darmstadt, Germany). Primary incubation was followed by incubation with secondary antibody after which the membranes were washed and incubated with the West Femto or West Pico luminal/enhancer solution and stable peroxide solution (Pierce, Rockford, IL, USA) before being exposed to an X-ray film (ThermoFisher Scientific, Waltham, MA, USA). The exposed films were subsequently processed using X-ray developing machine (Konica Minolta, Tokyo, Japan). All the experiments were performed at least in triplicates.

### Aβ_40_ ELISA Immunoassay

Cells were seeded at a density of 1 × 10^5^ cells/2 mL of medium in 6 well plates. 24 h post-seeding the cells were treated with 25 µM DHA or vehicle-control and the conditioned medium was collected from the cells after 24 and 48 h treatment duration. The collected conditioned medium was centrifuged and the cell-free supernatant was used for Aβ_40_ ELISA immunoassay using BetaMark™ x-40 ELISA Kit (Covance Inc., NJ, USA) as per manufacturer’s instructions.

### Sample Preparation and Derivatization

Cells were seeded at a density of 1 × 10^5^ cells/2 mL of medium in 6 well plates. 24 h post-seeding, 25 µM DHA was administered to the cells and DMSO (at a final concentration of 0.1% *v/v*) was used as the vehicle control. The optimal *in vitro* concentration of DHA was selected based on the lack of observed toxicity and no less than 80% cell survival in both the cell types. Treatment with both DHA and DMSO was terminated after 24 h by harvesting the culture medium and washing the cells twice with ice-cold PBS. The collected culture medium was centrifuged and the cell-free supernatant was stored immediately at −80°C until further analysis. After quenching the metabolism with 1 mL ice-cold methanol, cells were collected and stored at −80°C until further sample preparation. Six independent biological replicates were examined for each treatment group and all the samples were subjected to extraction process to collect cell free supernatants (supporting information – Text S1). The collected supernatants of both the medium and the lysate samples were then concentrated to complete dryness at 50°C under a gentle stream of nitrogen gas using the TurboVap LV (Caliper Life Science, Hopkinton, MA, USA), followed by an additional drying step after addition of 100 µL of anhydrous toluene (dried over anhydrous sodium sulfate) to ensure complete removal of water. The dried extract were then subjected to methoximation using 50 µL of MOX reagent (2 h at 60°C), followed by trimethylsilyl (TMS) derivatization using 100 µL of MSTFA with 1% TMCS as catalyst (1 h at 60°C). The formed TMS derivatives were cooled to room temperature (24±1°C) and transferred to the autosampler vials for GC/TOFMS analysis.

### GC/TOFMS Conditions

GC/TOFMS analysis was performed on an Agilent 7890A Gas Chromatography (Agilent Technologies, Santa Clara, CA, USA) coupled to a PEGASUS 4D Time-of-Flight Mass Spectrometer (LECO Corporation, St. Joseph, MI, USA). The primary column used was a DB-1 GC column (Agilent Technologies) of internal diameter of 250 µm, length of 23 m and film thickness of 0.25 µm. Helium was used as the carrier gas at a flow rate of 1 mL/min. The injection volume was 1 µL. A splitless injection was used for the cell media while a split ratio of 1∶2 was used for the cell lysate. The optimized GC/MS front inlet and ion source temperatures were 250 and 200°C, respectively. The oven temperature was kept at 70°C for 20.0 min, increased to 250°C at 8°C/min and finally to 300°C at 40°C/min where it remained for 6 min. The transfer line was maintained at 250°C. The MS was operated using an electron impact (EI) ionization source at −70 eV and a detector voltage of 1800 V. The MS data were acquired in scan mode over the range *m/z* 50–600 at a rate of 15 spectra/s. Peaks with signal-to-noise (S/N) ratio lower than 100 were rejected. Quality control (QC) samples were prepared by randomly pooling 40 µL from each of the six medium samples of the control group Alkane series C_8_–C_40_ and fatty acid methyl esters (FAME) (C_8_– C_28_) standards were analyzed with the same GC program for calculation of the retention index of the metabolites. LECO ChromaTOF software (version 4.21, LECO Corporation, St. Joseph, MI, USA) was used for chromatogram acquisition, peak deconvolution, analyte alignment and preliminary analyte identification by the National Institute of Standards and Technology (NIST) library (Wiley registry, NJ, USA), Fiehn library and internal spectral libraries. Based on the library matching, peaks with similarity index of more than 60% were given putative metabolite identities.

### Multivariate Data Analysis and Metabolite Identification

The peaks were normalized to the total integral area prior to chemometric and statistical data analysis. All processed data were mean centered and scaled to unit variance during chemometric data analysis. Principal component analysis (PCA) and partial least squares and discriminant analysis (PLS-DA) were employed to process the acquired and normalized data using SIMCA-P+ software (version 11, Umetrics, Umeå, Sweden). PCA score plots were used for observing the clustering trend among the samples as well as to detect and exclude outliers. After exclusion of outliers, medium and lysate samples were further subjected to PLS-DA for identifying models that differentiated groups or classes. The validity of the model was checked by performing 100 permutation tests. The criteria for model validity are as follows. First, all the *Q* ^2^ values on the permuted data set must be lower than the *Q* ^2^ value on the actual data set. Second, the regression line in validation plot (line joining the actual *Q* ^2^ point to the centroid of the cluster of permuted *Q* ^2^ values) must demonstrate a negative intercept on the *y-axis*. The validated models were subsequently utilized for identifying unique metabotypes associated with the DHA- and vehicle-treated groups. Variable importance in the projection (VIP) cutoff value was set as 1.00. Statistical comparison of discriminant metabolite levels between different groups was carried out using independent *t*-test with Welch’s correction; metabolites with a *p*-value of less than 0.05 being considered to be statistically significant. These putative marker metabolites were then cross-referenced against the Golm Metabolite Database (GMD) [21] and the Human Metabolome Database (HMDB) [22]. Identities of these metabolites were further ascertained by comparing their mass spectra and retention indices with that of commercially available reference standards Lastly, the Kyoto Encyclopaedia of Genes and Genomes (KEGG) [23] database was used for the interpretation of the metabolic pathways of the identified marker metabolites. Difference in the levels of metabolite between the treatment group was assessed by fold change values, where fold change  =  CHO−AβPP_695(treatment)_/CHO−wt_(treatment)_ and values >1 and <1 represented higher and lower metabolite levels observed in that treatment group, respectively.

### Pyruvate Dehydrogenase Enzyme Assay

Both CHO-wt and CHO-AβPP_695_ cells were seeded at a density of 1.5×10^6^/10 mL in a T_75_ flask followed by treatment with either 25 µM DHA or DMSO as vehicle control. The cells were harvested 24 h post treatment and lysed in lysis buffer containing 1% Triton with protease inhibitor. Each cell lysate mixture was centrifuged at 13000 *g* for 10 min at 4°C and protein concentration of the soluble extract was determined by Bradford protein assay. An aliquot of the supernatant was diluted with the lysis buffer to achieve a protein concentration of 400 µg/mL for each sample. The assay was conducted as per the manufacturer’s instructions with the final enzyme activity being assessed by measuring the reduction of NAD^+^ to NADH at 340 nm using Infinite® 200Pro spectrophotometer (Tecan, Crailsheim, Germany). Pyruvate dehydrogenase (PDH) enzyme concentration (mU/mL) was determined from the regression equation of the generated standard curve.
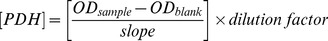



Statistical analysis was done via ANOVA with Bonferroni’s post-hoc analysis, with **p<0.01 and ***p<0.001 considered to be statistically significant.

## Results

### Cell Model Validation

**Figure 1:**
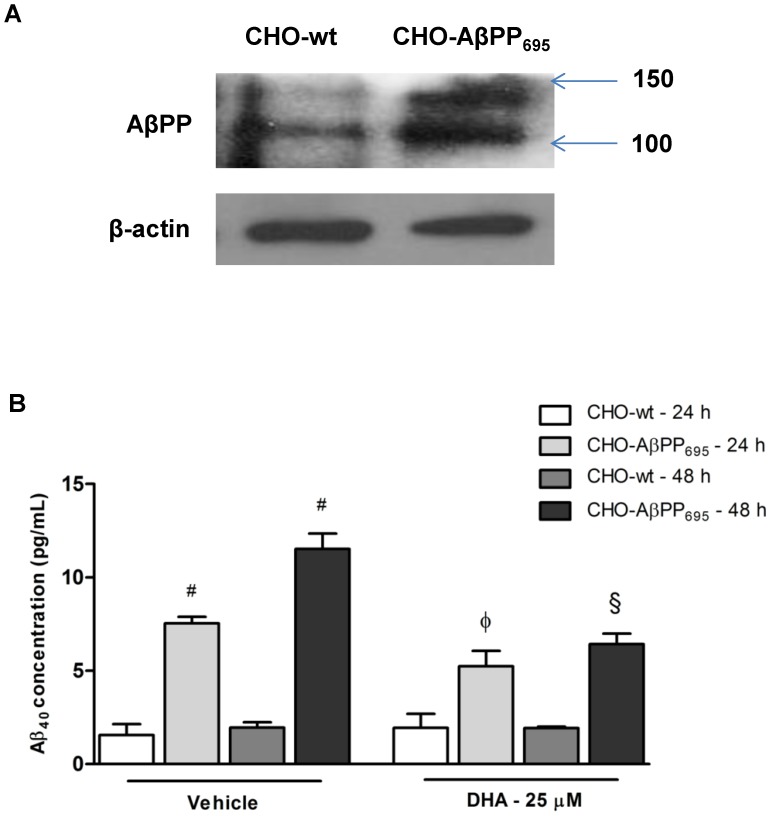
Model validation for CHO-wt and CHO-AβPP_695_ cells and effect of DHA on Aβ_40_ release. (A) Conditioned medium was collected from CHO-wt and CHO-AβPP_695_ cells with and without DHA treatment and subjected to ELISA immunoassays for Aβ_40._ There was negligible release of Aβ_40_ from CHO-wt cells as compared to CHO-AβPP_695_ cells at 24 and 48 h. A significant decrease was observed in the release of Aβ_40_ in CHO-AβPP_695_ cells after treatment with 25 µM DHA for 24 h and 48 h. ^#^ *p*<0.001 as compared to CHO-wt vehicle treated cells, ^φ^ *p*<0.05 compared to CHO-AβPP_695_ 24 h vehicle treatment and ^§^ *p*<0.001 as compared to CHO-AβPP_695_ 48 h vehicle treatment. Analysis was done via ANOVA with Bonferroni’s post-hoc analysis. (B) Western blot analysis of the cell lysates confirm AβPP_695_ plasmid overexpression in CHO-AβPP_695_ cells compared to CHO-wt.

AβPP are transmembrane glycoproteins that exist in three major isoforms, 770, 751 and 695, of which the 695 fragment containing 695 amino acids is predominantly expressed in neuronal cells [24]. Various *in-vitro* AD cell models have been developed overexpressing AβPP_695_ fragment for AD studies including CHO-AβPP_695_ [25]–[29]. To validate our cell model, western blot analysis was performed and confirmed the overexpression of AβPP_695_ protein in CHO-AβPP_695_ cells compared to the parental CHO-wt cells (Figure 1A). On the other hand, ELISA immunoassays showed that Aβ_40_ is released significantly only in the conditioned medium of CHO-AβPP_695_ cells, even after 48 h (Figure 1B). These results established the validity of our cell model for subsequent metabolomic analysis.

### GC/TOFMS Metabolic Profiling

**Figure 2:**
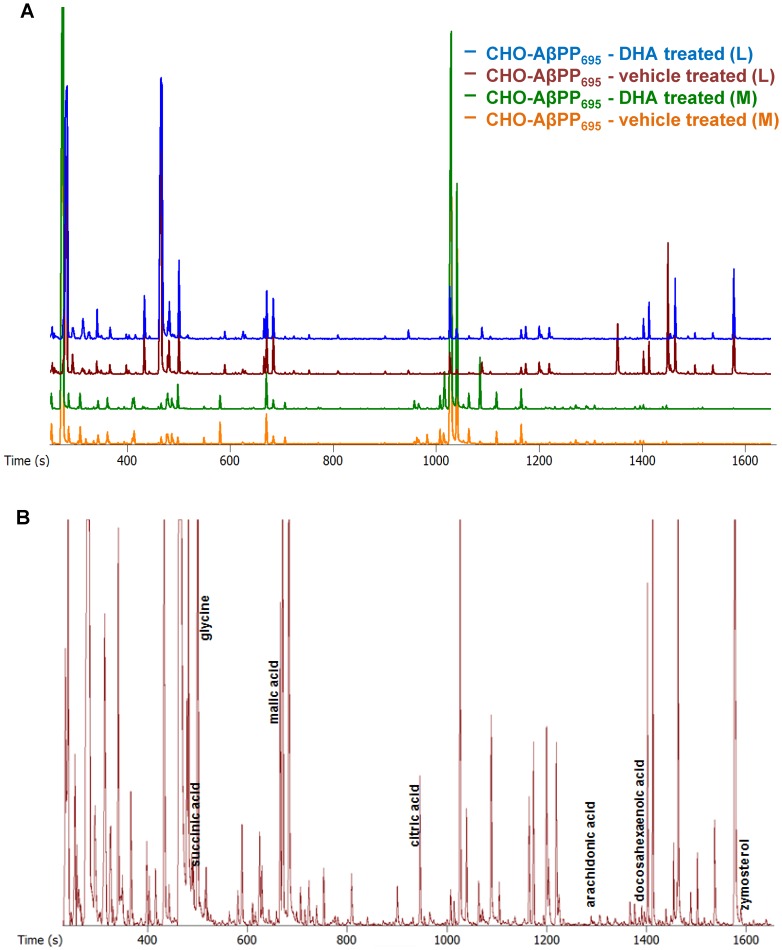
Overlay of GC/TOFMS chromatograms. (A) Representative GC/TOFMS chromatogram of DHA-treated and vehicle-treated CHO-AβPP_695_ cells – lysate (L) and medium (M) samples (B) Representative chromatogram demonstrating discriminatory metabolites between vehicle-treated and DHA-treated CHO-wt cells and CHO-AβPP_695_ cells.

Based on the survival curve (supporting information - Figure S1), 25 µM dose of DHA was selected for carrying out further analysis as it resulted in no significant toxicity and no less than 80% cell survival in both the cell types. Representative GC/TOFMS chromatogram of medium and lysate samples of DHA- and vehicle- treated CHO-AβPP_695_ cells is shown in Figure 2. A similar representative GC/TOFMS chromatogram of medium and lysate samples of DHA- and vehicle- treated CHO-wt cells is shown in supporting information - Figure S2. Chemometric data analysis revealed a distinct clustering trend between CHO-wt and CHO-AβPP_695_ cells treated with DHA and vehicle control in both the lysate and medium samples. For lysate samples, unsupervised PCA scores plots showed differences between the two cell types in vehicle-treated (R^2^X = 0.578 and Q^2^ (cum) = 0.295) and DHA-treated groups (R^2^X = 0.655 and Q^2^ (cum) = 0.432). A similar clustering was observed for medium samples in vehicle-treated (R^2^X = 0.990 and Q^2^ (cum) = 0.114) and DHA-treated groups (R^2^X = 0.681 and Q^2^ (cum) = 0.459).

**Figure 3:**
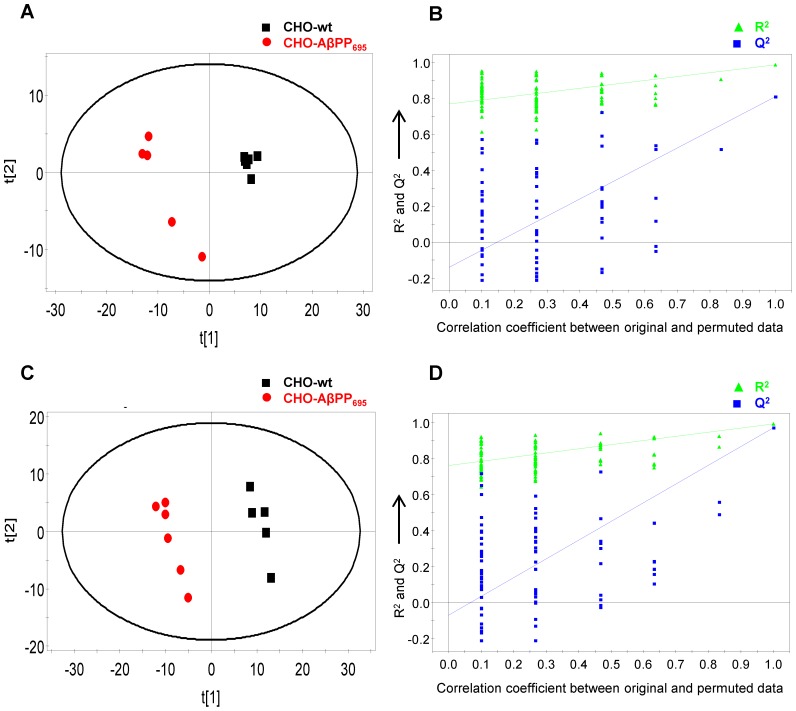
PLS-DA score plot and validation plot for lysate samples. (A) PLS-DA score plot of vehicle-treated CHO-wt and CHO-AβPP_695_ lysate samples (R^2^X = 0.474; R^2^Y = 0.985; Q^2^ (cum) = 0.808; LV = 2); (B) Validation plot of the PLS-DA model obtained from 100 permutation tests for vehicle-treated lysate samples; (C) PLS-DA score plot of DHA-treated CHO-wt and CHO-AβPP_695_ lysate samples (R^2^X = 0.645; R^2^Y = 0.993; Q^2^ (cum) = 0.971; LV = 2); (D) Validation plot of the PLS-DA model obtained from 100 permutation tests for DHA-treated lysate samples.

**Figure 4:**
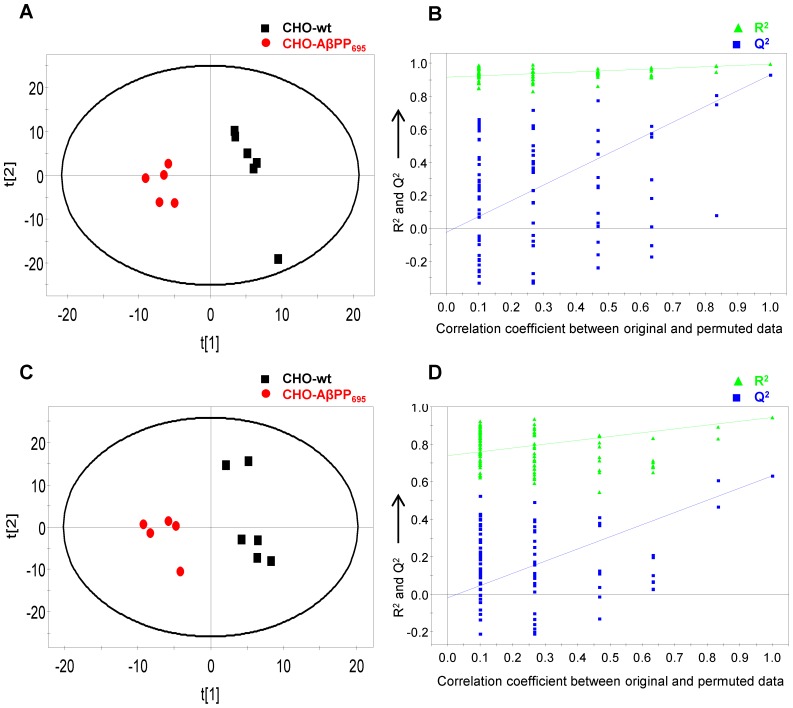
PLS-DA score plot and validation plot for medium samples. (A) PLS-DA score plot of vehicle-treated CHO-wt and CHO-AβPP_695_ medium samples (R^2^X = 0.679; R^2^Y = 0.994; Q^2^ (cum) = 0.929; LV = 3); (B) Validation plot of the PLS-DA model obtained from 100 permutation tests for vehicle-treated medium samples; (C) PLS-DA score plot of DHA-treated CHO-wt and CHO-AβPP_695_ medium samples (R^2^X = 0.745; R^2^Y = 0.992; Q^2^ (cum) = 0.885; LV = 3); (D) Validation plot of the PLS-DA model obtained from 100 permutation tests for DHA-treated medium samples.

Subsequent supervised PLS-DA model uncovered a list of putative marker metabolites that were significantly different (*p*<0.05 for T-test with Welch’s correction) and characterized the metabotypes associated with DHA treatment effect (Figure 3 and 4). PLS-DA model was optimized with 2 latent variables (LV) for lysate samples; vehicle-treated (R^2^X = 0.474, R^2^Y = 0.985 and Q^2^ (cum) = 0.808) and DHA-treated groups (R^2^X = 0.645, R^2^Y = 0.993 and Q^2^ (cum) = 0.971), whereas model for medium samples was optimized with 3 LV; vehicle-treated (R^2^X = 0.679, R^2^Y = 0.994 and Q^2^ (cum) = 0.929) and DHA-treated groups (R^2^X = 0.745, R^2^Y = 0.992 and Q^2^ (cum) = 0.885). All the PLS-DA models were found to be valid with no overfitting of data based on the criteria of the permutation test. A list of all the identified marker metabolites is summarized in supporting information - Table S1. A list of discriminant marker metabolites that have biological relevance in AD and significantly differentiated DHA-treated and vehicle-treated groups in PLS-DA model for both CHO-wt and CHO-AβPP_695_ cell types is summarized in Table 1.

**Table 1:** Discriminatory marker metabolites identified from medium and lysate samples of DHA-treated and vehicle-treated CHO-wt and CHO-AβPP_695_ cells.

Metabolite	Sample	Chemical class	Kovats RI	Vehicle-treated			DHA-treated		
				Normalized peak area (×10^−4^)^c^		Fold Δ^d^	Normalized peak area (×10^−4^)^c^		Fold Δ^d^
				CHO-wt	CHO-AβPP_695_		CHO-wt	CHO-AβPP_695_	
Citric acid^a^	lysate	TCA	1844.5	7.5±0.9	8.4±3.3*	1.12	16.6±1.5	33.0±2.4*	1.99
Malic acid^a^	lysate	TCA	1501.6	20.5±1.5	21.6±1.3^ns^	1.05	16.6±0.9	25.3±1.7*	1.52
DHA^a^	lysate	Fatty acid	2552.3	3.2±1.4	2.5±0.7*	0.79	3.5±0.2	4.5±0.3*	1.28
Arachidonic acid^a^	lysate	Fatty acid	2352.8	3.1±0.3	3.5±0.4*	1.17	3.2±0.1	2.7±0.3*	0.85
Zymosterol^b^	lysate	Steroid	3170.0	4.3±0.4	7.6±0.9*	1.76	4.2±0.3	6.6±0.7*	1.59
Cholesta-3,5-diene^b^	lysate	Steroid	2885.6	3.0±0.3	3.1±0.4^ ns^	1.05	3.0±0.4	1.5±0.5*	0.52
Succinic acid^a^	medium	TCA	1305.9	3.2±0.5	3.5±0.1^ns^	1.09	3.4±0.3	4.1±0.3*	1.20
Malic acid^a^	medium	TCA	1501.6	1.3±0.1	1.2±0.3^ns^	0.92	1.4±0.1	1.6±0.1*	1.17
Glycine^a^	medium	Amino acid	1316.1	80.8±20.9	102.1±29.4^ns^	1.30	181.4±58.3	284.6±47.6*	1.56

a Metabolite identification using standard compound.

b Metabolite identification using NIST library search.

c Normalized peak area values expressed as mean ± S.E.M.

d Fold change (Δ): CHO-AβPP_695 (treatment)/_CHO-wt _(treatment)_.

*p<0.05 and ^ns^ not significant when calculated using the independent *t*-test with Welch’s correction for normalized peak area of CHO-AβPP_695_ cells compared to CHO-wt cells for respective treatment groups.

Abbreviations: DHA – docosahexaenoic acid, TCA – tricarboxylic acid.

From Figure 1 and Table 1, we can see that even before sufficient release of Aβ_40_ in the culture medium (24 h versus 48 h), AβPP induced metabolic changes in CHO-AβPP_695_ cells as compared to CHO-wt cells. HMDB and KEGG databases were used to elucidate the pathways that were associated with the identified marker metabolites. With DHA treatment, the levels of citric acid, malic acid and DHA were found to be elevated while that of arachidonic acid, cholesta-3, 5-diene and zymosterol were found to be significantly lowered in the lysate samples (Table 1). These perturbed metabolites were found to correlate with metabolic pathways of the TCA cycle (citric acid and malic acid), cholesterol metabolism (zymosterol and cholesta-3, 5-diene) and fatty acid biosynthesis (DHA and arachidonic acid). Similarly, in medium samples, the levels of glycine, malic acid and succinic acid were found to be elevated in DHA-treated groups. The metabolic networks for these metabolites were correlated with specific metabolic pathways of tricarboxylic acid cycle (TCA) (succinic acid and malic acid) and amino acid metabolism (glycine). As temporal longitudinal metabolic flux measurements (fluxomics experiments) had not been performed in this study, neither the relationships between the lysate versus medium marker metabolites nor the rates of metabolic reactions in biological systems could be determined. Table 2 summarizes the findings about the marker metabolites, their associated metabolic pathways and their biological significance in AD. Our findings suggested that DHA possibly acts by mitigating AβPP-induced deregulations in TCA cycle, steroid and fatty acid biosynthesis.

**Table 2:** Metabolites, their associated metabolic pathways and biological relevance in AD.

Metabolites^a^	Metabolic pathway^b^	Biological relevance in AD
Citric acid (↓)	TCA cycle	Deregulation of TCA cycle, hypometabolism and increased oxidative damages
Succinic acid (↓)	TCA cycle	Deregulation of TCA cycle, hypometabolism and increased oxidative damages
Malic acid (↓)	TCA cycle	Deregulation of TCA cycle, hypometabolism and increased oxidative damages
Glycine (↓)	Amino acid metabolism	Required for synthesis of heme which is essential for functioning of electron transport chain
Zymosterol (↑)	Steroid biosynthesis	Increased risk of formation and deposition of amyloid beta plaques from APP
Arachidonic acid (↑)	Eicosanoid biosynthesis	Generation of pro-inflammatory and inflammatory mediators in AD
DHA (↓)	Fatty acid biosynthesis	Affects neuroprotection, successful aging, memory and inflammation resolving properties

a Metabolites are grouped together on the basis of their biological relevance. (↑) elevated in AD and (↓) reduced in AD.

b Related to metabolites using KEGG database.

Abbreviations: DHA – docosahexaenoic acid, TCA – tricarboxylic acid.

### Pyruvate Dehydrogenase Enzyme Assay

**Figure 5:**
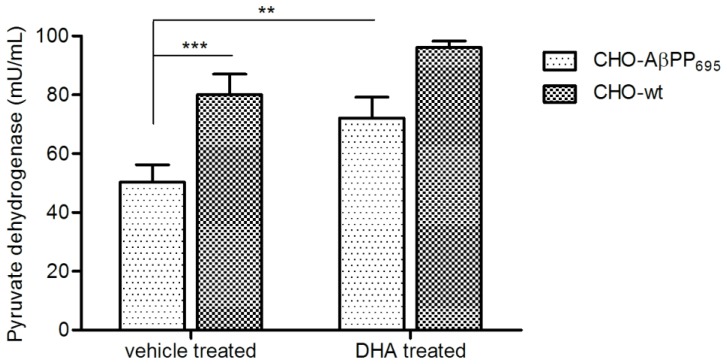
DHA treatment increases pyruvate dehydrogenase enzyme concentration in CHO-wt and CHO-AβPP_695_ cells. Pyruvate dehydrogenase activity in CHO-wt and CHO-AβPP_695_ cells treated with vehicle or 25 µM DHA. Values are means ± SEM from three independent experiments. **p<0.01 and ***p<0.001 as compared to CHO-AβPP_695_ vehicle treated. Analysis was done via ANOVA with Bonferroni’s post-hoc analysis.

PDH is a rate limiting enzyme connecting the glycolysis and TCA cycle and is found to be significantly decreased in AD patients. Similarly, as seen in Figure 5, PDH enzyme concentration (mU/mL) was observed to be significantly lower in vehicle treated CHO-AβPP_695_ compared to CHO-wt cells (50.3±5.9 vs. 80.5±7.1, ***p<0.001) in our study. This decrease in PDH enzyme activity in CHO-AβPP_695_ confirmed the involvement of AβPP in perturbing mitochondrial enzymatic activities and provided an additional validation for the use of these cells as surrogate models for the diseased and healthy patients. DHA treatment for 24 h increased the enzyme concentration in both CHO-wt (96.1±2.2) and CHO-AβPP_695_ (72.1±7.1) cells as compared to their respective vehicle controls. This increase in enzyme concentration in DHA-treated CHO-AβPP_695_ cells was statistically significant as compared to vehicle-treated CHO-AβPP_695_ cells (72.1±7.1 vs. 50.3±5.9, **p<0.01).

### Effect of DHA on Aβ_40_ Release

Cleavage of AβPP by β- and γ-secretases yields numerous Aβ fragments, of which Aβ_40_ and Aβ_42_ are the most toxic. Among the two, Aβ_40_ has been found to be 10-fold higher in abundance than Aβ_42_ in patients with sporadic AD [30]. As reported by other studies [31]–[33], DHA treatment has been shown to reduce the release of Aβ from its precursor, AβPP. In order to evaluate the biological effects of DHA, we also undertook a similar experiment wherein we evaluated the effect of DHA on the release of Aβ_40_ from CHO-AβPP_695_ cells. As seen from Figure 1B, release of Aβ_40_ was significantly higher at both 24 and 48 h time duration as compared to CHO-wt cells, with the release at 48 h time duration being comparatively higher than that at 24 h. DHA treatment at 25 µM concentration was able to decrease the release of Aβ_40_ from CHO-AβPP_695_ cells at both 24 and 48 h treatment durations by approximately 50%. In addition to the metabolomics data, this result further corroborated the effect of DHA in reducing AβPP mediated release of Aβ peptides and the ensuing toxic damages to the cells.

## Discussion

Mitochondrial dysfunctions and associated energy hypometabolism have been considered as one of the important factors contributing to the etiology of AD. Significant amount of work has been done by various research groups showing the deleterious effects of Aβ on the mitochondrial activity. However, it has been suggested recently that AβPP, the parent precursor to Aβ, is toxic and affects cellular metabolic activities. Trafficking of AβPP in the mitochondrial matrix has been reported to impair functions of various mitochondrial enzymes and leading to disturbances in energy metabolism [13], [34]. Amongst the affected enzymes of TCA cycle, activity of α-ketoglutarate dehydrogenase, pyruvate dehydrogenase and isocitrate dehydrogenase have been reported to be markedly decreased in autopsied brains of AD patients while the activity of succinate dehydrogenase and malate dehydrogenase have been shown to be markedly increased [35]. These enzyme alterations result in a reduction of succinyl CoA via its rapid conversion to other metabolites that in turn reduces the production of heme, a porphyrin molecule necessary for maintaining the integrity of the ETC. This net decrease in the production of ATP molecules establishes a state of hypometabolism and oxidative stress in an AD brain.

Based on the results of pre-clinical and clinical studies, the therapeutic effects of DHA and its isolated metabolites in mitigating Aβ- induced neurodegeneration, memory impairment and inflammation have been established [10], [11], [36]–[39]. DHA has also been shown to reduce the generation of Aβ from its precursor AβPP [31] and we have observed similar results in our study. Through this study we have attempted to further characterize the mechanism by which DHA mitigates AβPP-induced metabolic changes by profiling the metabotypes associated with DHA-treated and untreated wild type and AβPP_695_ cell models.

### Effect of DHA on AβPP Impaired Energy Metabolism Pathways

GC/TOFMS characterization of CHO-wt and CHO-AβPP_695_ at 24 h yielded distinct metabotypes associated with the DHA-treated CHO-AβPP_695_ cells. With DHA treatment, we found elevated levels of citric acid, succinic acid and malic acid in CHO-AβPP_695_ cells suggesting a possible mitigation of AβPP-perturbed TCA enzyme activities commonly observed in AD patients. Upregulation of the decreased PDH enzyme level in CHO-AβPP_695_ cells to the basal level of CHO-wt cells further corroborated the DHA-effected changes in the metabolite levels. In addition, level of glycine was also found to be elevated upon DHA treatment. Glycine, an amino acid, is required along with succinyl-CoA for the synthesis of porphyrins and efficient functioning of the ETC. Our findings suggested that DHA possibly mitigates AβPP-suppressed mitochondrial activity and associated hypometabolism of the CHO-AβPP_695_ cells, thus enabling them to recover from the metabolic perturbations.

### Effect of DHA on Cholesterol Metabolism

There is a growing consensus underlying the mechanistic link between the formation of amyloid plaques and cholesterol metabolism [40]. Even though the effect of cholesterol on production of Aβ and metabolism is not yet completely defined, Barrett *et al* recently reported that APP has a flexible transmembrane domain that can bind cholesterol and form an avid complex leading to enhanced amyloidogenesis [41]. It is also believed that the membrane cholesterol content serves as a pro-amyloidogenic factor, governing the activity of the enzymes playing a role in amyloidogenic processing of AβPP to Aβ [42]–[45]. Apart from cholesterol, the oxidized products of cholesterol called oxysterols have also been studied for their possible role in the disease pathogenesis, due to their involvement in cholesterol regulation. In addition, *in vivo* studies carried out using statins [42] and other cholesterol lowering drugs in AD models have reported the therapeutic effect of lowering cholesterol content in diminishing Aβ deposition and Aβ load of the brain [46], [47]. Increased consumption of PUFAs (notably the omega-3 family) has been associated with the reduction of risk in developing AD by lowering cholesterol levels. Levels of zymosterol, a precursor to cholesterol synthesis, and cholesta-3, 5-diene, an oxysterol, were found to be reduced in CHO-AβPP_695_ cells as compared to CHO-wt with DHA treatment. As acetyl CoA is a common precursor and link between the TCA cycle and the cholesterol biosynthesis pathway, the question whether more acetyl CoA is being diverted towards energy metabolism pathways in the presence of DHA and thus reducing its availability for cholesterol synthesis, still remains to be answered. These results show that DHA plays a role in reducing cholesterol biosynthesis which is a potentially AD-preventive mechanism as demonstrated by cholesterol-lowering statins.

### Effect of DHA on Fatty Acid Metabolism

The possible contribution of inflammation in the etiology and pathogenesis of AD has been questioned over for many years now. Several pharmacological approaches have been proposed to control these inflammatory responses in order to prevent destruction of the viable tissues in an AD brain. Arachidonic acid, an omega-6 fatty acid, precursor to various pro-inflammatory and inflammatory molecules, has been postulated to be an important mediator of inflammation related damages in AD [48]–[50]. Arachidonic acid mediated inflammation has been reported to induce tau protein polymerization [51] and enhance generation of reactive oxygen species. A direct role of arachidonic acid in the pathogenesis of AD was highlighted in a study conducted using positron emission tomography (PET) on brains of live, non-anaesthetized humans [52], where compared to their age matched controls, an elevated arachidonic acid metabolism was reported in AD patients. On the contrary, endogenous level of DHA was found to be significantly reduced in the brains of AD patients. As DHA and its metabolites have been known to exhibit anti-inflammatory activities, this reduction in the levels of DHA in AD patients may lead to further aggravation of the inflammation mediated damages in AD brain.

We found that treatment with DHA resulted in a reduction in the endogenous levels of arachidonic acid in CHO-AβPP_695_ cells as compared to CHO-wt. On the other hand, DHA levels were found to be increased in CHO-AβPP_695_ cells post-treatment. This increase in DHA levels, however, can be attributed to the exogenous DHA treatment of the cells. Both omega-3 and omega-6 fatty acids share the same biosynthetic pathway and compete for pathway enzymes and incorporation into the cell membranes. Dietary consumption of DHA would result in a displacement of arachidonic acid from the cell membranes and serve as a precursor for the synthesis of anti-inflammatory metabolites, such as resolvins and protectins. Since enzymatic metabolism of arachidonic acid generates inflammatory mediators, decreased levels of arachidonic acid would also imply a decrease in the generation of its downstream metabolites and thus, a reduction in the damages mediated by ensuing inflammation and oxidative stress.

## Conclusion

GC/TOFMS-based metabonomic profiling approach served as a sensitive and powerful tool for identification of discriminant metabolites associated with control and DHA-treated CHO-wt and CHO-AβPP_695_ cells. The metabolites identified were mainly involved in TCA cycle, cholesterol metabolism pathway and arachidonic acid metabolism. Our study provides an understanding of the metabolic changes induced by AβPP trafficking in the mitochondrial matrix and the mechanism by which DHA helps in mitigating these disturbances and restores the metabolic homeostasis of the mitochondria at an early stage. In addition, the preliminary effect of DHA on cholesterol metabolism also fuels the need to dive deeper into the mechanistic effects of DHA on the cholesterol metabolism pathway. Considering DHA is clinically effective in improving cognitive functions at an early stage of AD [36], [53] it may become a potential prophylactic agent for delaying the progression of this multi-factorial disease. In the same light, our findings will provide concurrence insights towards conceptualizing effective therapeutic outcomes using DHA for the prophylaxis of this devastating disorder.

## Supporting Information

Figure S1 **Concentration-dependent effect of DHA on CHO-wt and CHO-AβPP_695_ cell viability over 24 h.** The data as derived from three independent MTT experiments repeated in triplicate is presented as mean ± S.E.M relative to DMSO (vehicle control).
(DOCX)

Figure S2 **Overlay of representative GC/TOFMS chromatogram.** Representative GC/TOFMS chromatogram of DHA-treated and vehicle-treated CHO-wt cells – lysate (L) and medium (M) samples.
(DOCX)

Table S1 **Marker metabolites identified from medium and lysate samples of DHA-treated and vehicle-treated CHO-wt and CHO-AβPP_695_ cells.**
(DOCX)

Text S1 **Methods for Sample Preparation and Derivatization.**
(DOCX)

## References

[1] Florent-Béchard S, Malaplate-Armand C, Koziel V, Kriem B, Olivier J-L, et al (2007) Towards a nutritional approach for prevention of Alzheimer’s disease: Biochemical and cellular aspects. Journal of the Neurological Sciences 262: 27–36. 17681547 10.1016/j.jns.2007.06.046

[2] Ruxton CHS, Reed SC, Simpson MJA, Millington KJ (2004) The health benefits of omega-3 polyunsaturated fatty acids: a review of the evidence. Journal of Human Nutrition and Dietetics 17: 449–459. 15357699 10.1111/j.1365-277X.2004.00552.x

[3] Muskiet FAJ, van Goor SA, Kuipers RS, Velzing-Aarts FV, Smit EN, et al (2006) Long-chain polyunsaturated fatty acids in maternal and infant nutrition. Prostaglandins, leukotrienes, and essential fatty acids 75: 135–144. 10.1016/j.plefa.2006.05.010 16876396

[4] Bazan NG, Molina MF, Gordon WC (2011) Docosahexaenoic acid signalolipidomics in nutrition: Significance in aging, neuroinflammation, macular degeneration, Alzheimer’s, and other neurodegenerative diseases. Annual Review of Nutrition 31: 321–351. 10.1146/annurev.nutr.012809.104635 PMC3406932 21756134

[5] Kyle DJ, Schaefer E, Patton G, Beiser A (1999) Low serum docosahexaenoic acid is a significant risk factor for Alzheimer’s dementia. Lipids 34: S245–S245. 10419166 10.1007/BF02562306

[6] Mao P (2013) Oxidative stress and its clinical applications in dementia. Journal of Neurodegenerative Diseases 2013: 15. 10.1155/2013/319898 PMC4437276 26316986

[7] Serhan CN (2009) Systems approach to inflammation resolution: identification of novel anti-inflammatory and pro-resolving mediators. Journal of Thrombosis and Haemostasis 7: 44–48. 19630766 10.1111/j.1538-7836.2009.03396.x

[8] Lim GP, Calon F, Morihara T, Yang F, Teter B, et al (2005) A diet enriched with the omega-3 fatty acid docosahexaenoic acid reduces amyloid burden in an aged Alzheimer mouse model. The Journal of Neuroscience 25: 3032–3040. 15788759 10.1523/JNEUROSCI.4225-04.2005 PMC6725084

[9] Calon F, Lim GP, Yang F, Morihara T, Teter B, et al (2004) Docosahexaenoic acid protects from dendritic pathology in an Alzheimer’s disease mouse model. Neuron 43: 633–645. 15339646 10.1016/j.neuron.2004.08.013 PMC2442162

[10] Hashimoto M, Hossain S, Agdul H, Shido O (2005) Docosahexaenoic acid-induced amelioration on impairment of memory learning in amyloid β-infused rats relates to the decreases of amyloid β and cholesterol levels in detergent-insoluble membrane fractions. Biochimica et Biophysica Acta (BBA) - Molecular and Cell Biology of Lipids 1738: 91–98. 16427803 10.1016/j.bbalip.2005.11.011

[11] Hashimoto M, Tanabe Y, Fujii Y, Kikuta T, Shibata H, et al (2005) Chronic administration of docosahexaenoic acid ameliorates the impairment of spatial cognition learning ability in amyloid β–infused rats. The Journal of Nutrition 135: 549–555. 15735092 10.1093/jn/135.3.549

[12] Anandatheerthavarada HK, Devi L (2007) Amyloid precursor protein and mitochondrial dysfunction in Alzheimer’s disease. The Neuroscientist 13: 626–638. 17911214 10.1177/1073858407303536

[13] Devi L, Prabhu BM, Galati DF, Avadhani NG, Anandatheerthavarada HK (2006) Accumulation of amyloid precursor protein in the mitochondrial import channels of human Alzheimer’s disease brain is associated with mitochondrial dysfunction The Journal of Neuroscience. 26: 9057–9068. 10.1523/JNEUROSCI.1469-06.2006 PMC6675337 16943564

[14] Mao P, Reddy PH (2011) Aging and amyloid beta-induced oxidative DNA damage and mitochondrial dysfunction in Alzheimer’s disease: Implications for early intervention and therapeutics. Biochimica et Biophysica Acta (BBA) - Molecular Basis of Disease 1812: 1359–1370. 21871956 10.1016/j.bbadis.2011.08.005 PMC3185172

[15] Reddy PH (2006) Amyloid precursor protein-mediated free radicals and oxidative damage: implications for the development and progression of Alzheimer’s disease. Journal of Neurochemistry 96: 1–13. 10.1111/j.1471-4159.2005.03530.x 16305625

[16] Reddy PH, Manczak M, Mao P, Calkins MJ, Reddy AP, et al.. (2010) Amyloid-β and mitochondria in aging and Alzheimer’s disease: Implications for synaptic damage and cognitive decline. Journal of Alzheimers Disease 20 S499–S512. 10.3233/JAD-2010-100504 PMC3059092 20413847

[17] Reddy PH (2009) Amyloid beta, mitochondrial structural and functional dynamics in Alzheimer’s disease. Experimental Neurology 218: 286–292. 19358844 10.1016/j.expneurol.2009.03.042 PMC2710427

[18] Manczak M, Anekonda TS, Henson E, Park BS, Quinn J, et al (2006) Mitochondria are a direct site of Aβ accumulation in Alzheimer’s disease neurons: implications for free radical generation and oxidative damage in disease progression. Human Molecular Genetics 15: 1437–1449. 16551656 10.1093/hmg/ddl066

[19] Chan ECY, Pasikanti KK, Nicholson JK (2011) Global urinary metabolic profiling procedures using gas chromatography-mass spectrometry. Nature Protocols 6: 1483–1499. 21959233 10.1038/nprot.2011.375

[20] Ma Q-H, Futagawa T, Yang W-L, Jiang X-D, Zeng L, et al (2008) A TAG1-APP signalling pathway through Fe65 negatively modulates neurogenesis. Nature Cell Biology 10: 283–294. 18278038 10.1038/ncb1690

[21] Kopka J, Schauer N, Krueger S, Birkemeyer C, Usadel B, et al (2005) GMD@CSB.DB: the Golm Metabolome Database. Bioinformatics. 21: 1635–1638. 10.1093/bioinformatics/bti236 15613389

[22] Wishart DS, Tzur D, Knox C, Eisner R, Guo AC, et al (2007) HMDB: the human metabolome database. Nucleic Acids Research 35: D521–D526. 17202168 10.1093/nar/gkl923 PMC1899095

[23] Pedro M (2002) Emerging bioinformatics for the metabolome. Briefings in Bioinformatics 3: 134–145. 12139433 10.1093/bib/3.2.134

[24] Zhang YW, Thompson R, Zhang H, Xu H (2011) APP processing in Alzheimer’s disease. Molecular Brain 4: 1756–6606. 10.1186/1756-6606-4-3 PMC3022812 21214928

[25] Chen K-p, Dou F (2012) Selective interaction of amyloid precursor protein with different isoforms of neural cell adhesion molecule. Journal of Molecular Neuroscience 46: 203–209. 21691800 10.1007/s12031-011-9578-3

[26] Lakshmana MK, Yoon I-S, Chen E, Bianchi E, Koo EH, et al (2009) Novel role of RanBP9 in BACE1 processing of amyloid precursor protein and amyloid β peptide generation. Journal of Biological Chemistry 284: 11863–11872. 19251705 10.1074/jbc.M807345200 PMC2673255

[27] Shioi J, Georgakopoulos A, Mehta P, Kouchi Z, Litterst CM, et al (2007) FAD mutants unable to increase neurotoxic Aβ 42 suggest that mutation effects on neurodegeneration may be independent of effects on Aβ. Journal of Neurochemistry 101: 674–681. 17254019 10.1111/j.1471-4159.2006.04391.x

[28] Zhou S, Zhou H, Walian PJ, Jap BK (2005) CD147 is a regulatory subunit of the γ-secretase complex in Alzheimer’s disease amyloid β-peptide production. Proceedings of National Academy of Sciences USA 102: 7499–7504. 10.1073/pnas.0502768102 PMC1103709 15890777

[29] Phiel CJ, Wilson CA, Lee VMY, Klein PS (2003) GSK-3[alpha] regulates production of Alzheimer’s disease amyloid-[beta] peptides. Nature 423: 435–439. 12761548 10.1038/nature01640

[30] Näslund J, Schierhorn A, Hellman U, Lannfelt L, Roses AD, et al (1994) Relative abundance of Alzheimer A beta amyloid peptide variants in Alzheimer disease and normal aging. Proceedings of National Academy of Sciences USA 91: 8378–8382. 10.1073/pnas.91.18.8378 PMC44609 8078890

[31] Oksman M, Iivonen H, Hogyes E, Amtul Z, Penke B, et al (2006) Impact of different saturated fatty acid, polyunsaturated fatty acid and cholesterol containing diets on beta-amyloid accumulation in APP/PS1 transgenic mice. Neurobiology of Disease 23: 563–572. 16765602 10.1016/j.nbd.2006.04.013

[32] Lim GP, Calon F, Morihara T, Yang F, Teter B, et al (2005) A diet enriched with the omega-3 fatty acid docosahexaenoic acid reduces amyloid burden in an aged Alzheimer mouse model. Journal of Neuroscience 25: 3032–3040. 15788759 10.1523/JNEUROSCI.4225-04.2005 PMC6725084

[33] Grimm MO, Kuchenbecker J, Grosgen S, Burg VK, Hundsdorfer B, et al (2011) Docosahexaenoic acid reduces amyloid beta production via multiple pleiotropic mechanisms. Journal of Biological Chemistry 286: 14028–14039. 21324907 10.1074/jbc.M110.182329 PMC3077603

[34] Anandatheerthavarada HK, Biswas G, Robin M-A, Avadhani NG (2003) Mitochondrial targeting and a novel transmembrane arrest of Alzheimer’s amyloid precursor protein impairs mitochondrial function in neuronal cells. The Journal of Cell Biology 161: 41–54. 12695498 10.1083/jcb.200207030 PMC2172865

[35] Bubber P, Haroutunian V, Fisch G, Blass JP, Gibson GE (2005) Mitochondrial abnormalities in Alzheimer brain: Mechanistic implications. Annals of Neurology 57: 695–703. 15852400 10.1002/ana.20474

[36] Freund-Levi Y, Eriksdotter-Jonhagen M, Cederholm T, Basun H, Faxen-Irving G, et al (2006) Omega-3 fatty acid treatment in 174 patients with mild to moderate Alzheimer disease: OmegAD study: a randomized double-blind trial. Archives of Neurology 63: 1402–1408. 17030655 10.1001/archneur.63.10.1402

[37] Labrousse VF, Nadjar A, Joffre C, Costes L, Aubert A, et al (2012) Short-term long chain omega3 diet protects from neuroinflammatory processes and memory impairment in aged mice. PLoS ONE 7: e36861. 22662127 10.1371/journal.pone.0036861 PMC3360741

[38] Stonehouse W, Conlon CA, Podd J, Hill SR, Minihane AM, et al (2013) DHA supplementation improved both memory and reaction time in healthy young adults: a randomized controlled trial. The American Journal of Clinical Nutrition 97: 1134–1143. 23515006 10.3945/ajcn.112.053371

[39] Fotuhi M, Mohassel P, Yaffe K (2009) Fish consumption, long-chain omega-3 fatty acids and risk of cognitive decline or Alzheimer disease: a complex association. Nature Clinical Practice Neurology 5: 140–152. 10.1038/ncpneuro1044 19262590

[40] Shobab LA, Hsiung G-YR, Feldman HH (2005) Cholesterol in Alzheimer’s disease. The Lancet Neurology 4: 841–852. 16297842 10.1016/S1474-4422(05)70248-9

[41] Barrett PJ, Song Y, Van Horn WD, Hustedt EJ, Schafer JM, et al (2012) The amyloid precursor protein has a flexible transmembrane domain and binds cholesterol. Science 336: 1168–1171. 22654059 10.1126/science.1219988 PMC3528355

[42] Fassbender K, Simons M, Bergmann C, Stroick M, Lütjohann D, et al (2001) Simvastatin strongly reduces levels of Alzheimer’s disease beta-amyloid peptides Abeta 42 and Abeta 40 in vitro and in vivo. Proceedings of National Academy of Sciences USA 98: 5856–5861. 10.1073/pnas.081620098 PMC33303 11296263

[43] Bodovitz S, Klein WL (1996) Cholesterol modulates alpha-secretase cleavage of amyloid precursor protein. Journal of Biological Chemistry 271: 4436–4440. 8626795 10.1074/jbc.271.8.4436

[44] Frears ER, Stephens DJ, Walters CE, Davies H, Austen BM (1999) The role of cholesterol in the biosynthesis of beta-amyloid. Neuroreport 10: 1699–1705. 10501560 10.1097/00001756-199906030-00014

[45] Petanceska SS, DeRosa S, Olm V, Diaz N, Sharma A, et al (2002) Statin therapy for Alzheimer’s disease. Journal of Molecular Neuroscience 19: 155–161. 12212773 10.1007/s12031-002-0026-2

[46] Kandiah N, Feldman HH (2009) Therapeutic potential of statins in Alzheimer’s disease. Journal of the Neurological Sciences 283: 230–234. 19321181 10.1016/j.jns.2009.02.352

[47] Refolo LM, Pappolla MA, LaFrancois J, Malester B, Schmidt SD, et al (2001) A cholesterol-lowering drug reduces β-amyloid pathology in a transgenic mouse model of Alzheimer’s disease. Neurobiology of Disease 8: 890–899. 11592856 10.1006/nbdi.2001.0422

[48] Davies P, Bailey PJ, Goldenberg MM, Ford-Hutchinson AW (1984) The role of arachidonic acid oxygenation products in pain and inflammation. Annual Review of Immunology 2: 335–357. 10.1146/annurev.iy.02.040184.002003 6100476

[49] Samuelsson B (1983) Leukotrienes: mediators of immediate hypersensitivity reactions and inflammation. Science (New York, NY) 220: 568–575. 10.1126/science.6301011 6301011

[50] Kuehl FA, Egan RW (1980) Prostaglandins, arachidonic acid, and inflammation. Science (New York, NY) 210: 978–984. 10.1126/science.6254151 6254151

[51] Wilson DM, Binder LI (1997) Free fatty acids stimulate the polymerization of tau and amyloid beta peptides. In vitro evidence for a common effector of pathogenesis in Alzheimer’s disease. The American Journal of Pathology 150: 2181–2195. 9176408 PMC1858305

[52] Esposito G, Giovacchini G, Liow J-S, Bhattacharjee AK, Greenstein D, et al (2008) Imaging neuroinflammation in Alzheimer disease with radiolabeled arachidonic acid and PET. Journal of Nuclear Medicine 49: 1414–1421. 18703605 10.2967/jnumed.107.049619 PMC2587283

[53] Quinn JF, Raman R, Thomas RG, Yurko-Mauro K, Nelson EB, et al (2010) Docosahexaenoic acid supplementation and cognitive decline in Alzheimer disease. JAMA: the journal of the American Medical Association 304: 1903–1911. 21045096 10.1001/jama.2010.1510 PMC3259852

