# Supplementary material for: Metabotyping of Docosahexaenoic Acid - Treated Alzheimer’s Disease Cell Model
Source: PLoS One. 2014 Feb 27;9(2):e90123. doi: 10.1371/journal.pone.0090123 (PMC3937442; doi:10.1371/journal.pone.0090123)
Supplement: Figure S1 — Concentration-dependent effect of DHA on CHO-wt and CHO-AβPP695 cell viability over 24 h. The data as derived from three independent MTT experiments repeated in triplicate is presented as mean ± S.E.M relative to DMSO (vehicle control). (DOCX) [file pone.0090123.s001.docx]

**Supporting information - Figure S1**


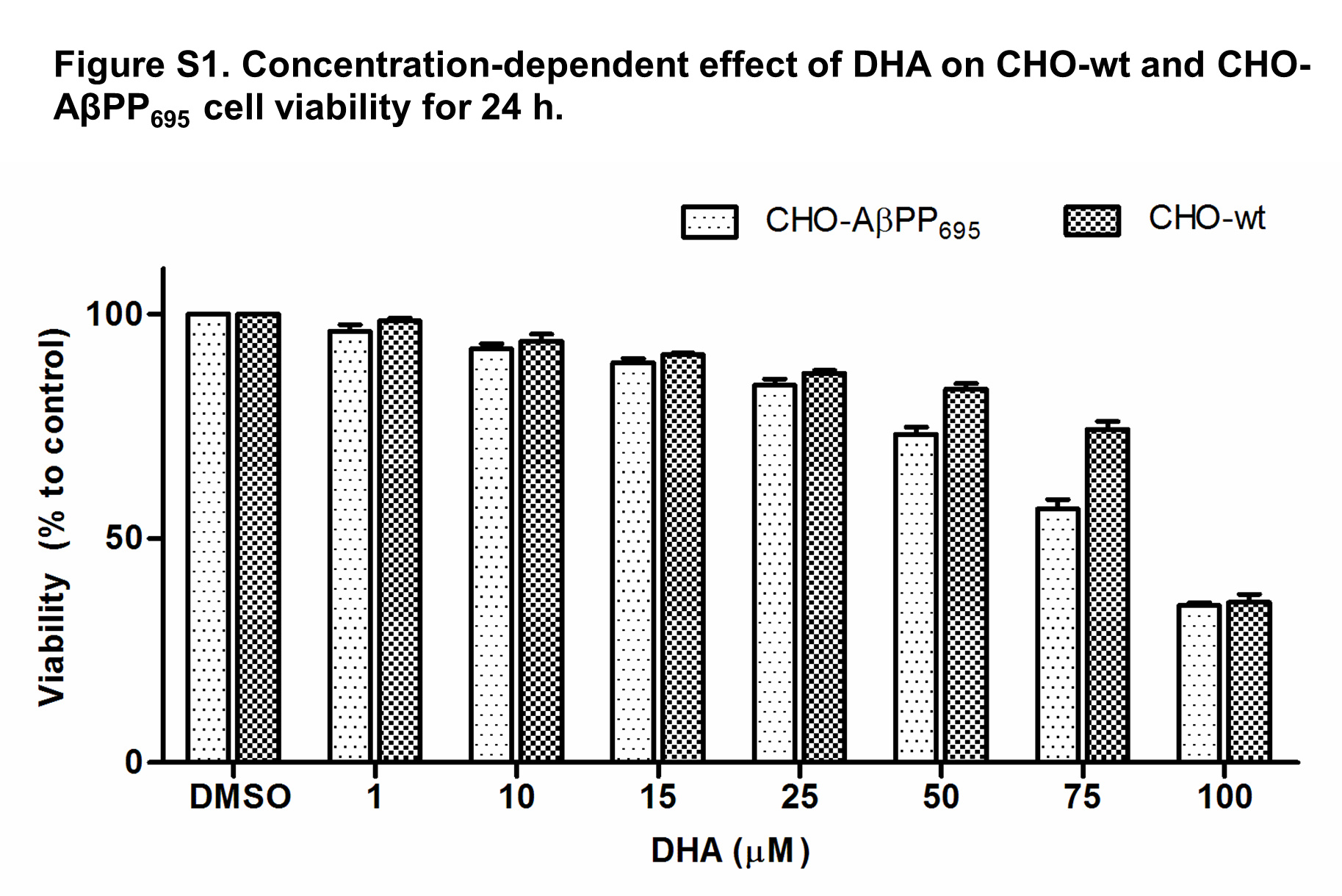


**Figure S1: Concentration-dependent effect of DHA on CHO-wt and CHO-AβPP_695_ cell viability over 24 h.** The data as derived from three independent MTT experiments repeated in triplicate is presented as mean ± S.E.M relative to DMSO (vehicle control).
