# Supplementary material for: Metabotyping of Docosahexaenoic Acid - Treated Alzheimer’s Disease Cell Model
Source: PLoS One. 2014 Feb 27;9(2):e90123. doi: 10.1371/journal.pone.0090123 (PMC3937442; doi:10.1371/journal.pone.0090123)
Supplement: Figure S2 — Overlay of representative GC/TOFMS chromatogram. Representative GC/TOFMS chromatogram of DHA-treated and vehicle-treated CHO-wt cells – lysate (L) and medium (M) samples. (DOCX) [file pone.0090123.s002.docx]

**Supporting information - Figure S2**


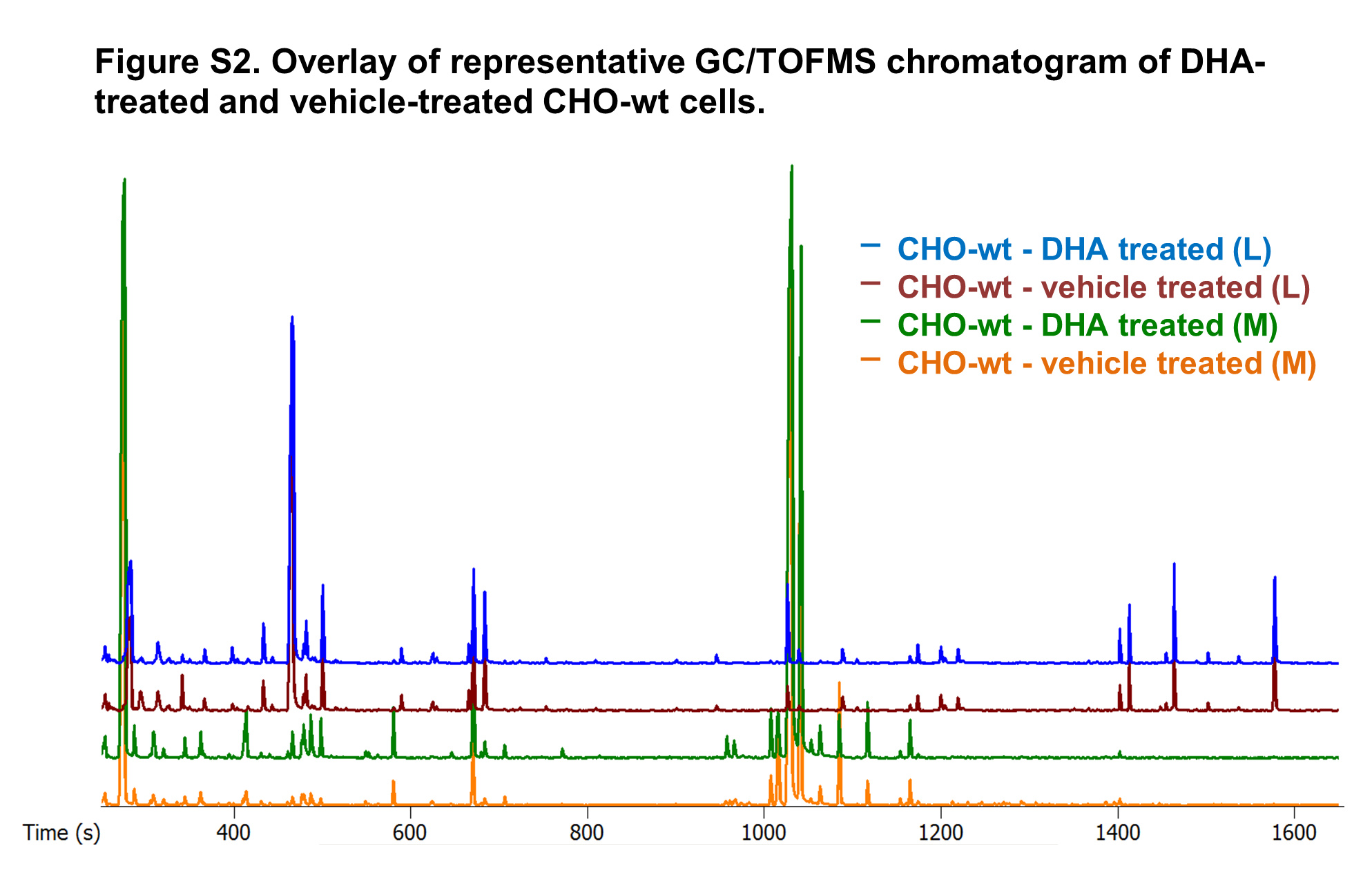


**Figure S2: Overlay of representative GC/TOFMS chromatogram.** Representative GC/TOFMS chromatogram of DHA-treated and vehicle-treated CHO-wt cells – lysate (L) and medium (M) samples**.**
