# Supplementary material for: Metabotyping of Docosahexaenoic Acid - Treated Alzheimer’s Disease Cell Model
Source: PLoS One. 2014 Feb 27;9(2):e90123. doi: 10.1371/journal.pone.0090123 (PMC3937442; doi:10.1371/journal.pone.0090123)
Supplement: Table S1 — Marker metabolites identified from medium and lysate samples of DHA-treated and vehicle-treated CHO-wt and CHO-AβPP695 cells. (DOCX) [file pone.0090123.s003.docx]

**Supporting information - Table S1: Marker metabolites identified from medium and lysate samples of DHA-treated and vehicle-treated CHO-wt and CHO-AβPP_695_ cells.**

| **Metabolite^a^** | **Sample** | **Kovats RI** | **Vehicle-treated** | | | **DHA-treated** | | |
| --- | --- | --- | --- | --- | --- | --- | --- | --- |
|  |  |  | **Normalized peak area(x 10^-4^)^b^** | | **Fold Δ^c^** | **Normalized peak area (x 10^-4^)^b^** | | **Fold Δ^c^** |
|  |  |  | **CHO-wt** | **CHO-AβPP_695_** |  | **CHO-wt** | **CHO-AβPP_695_** |  |
| Propanoic acid | medium | 1032.0 | 97.6 ± 22.1 | 131.2 ± 11.2 ^*^ | 1.34 | 83.0 ± 26.3 | 126.0 ± 16.6^*^ | 1.52 |
| Lactic acid | medium | 1059.6 | 902.3 ± 10.4 | 1371.1 ± 49.1^*^ | 1.52 | 844.5 ± 94.6 | 1157.8 ± 36.1^ns^ | 1.37 |
| Alanine | medium | 1100.2 | 280.3 ± 48.7 | 465.8 ± 70.1^*^ | 1.66 | 345.5 ± 75.2 | 568.9 ± 86.5^*^ | 1.65 |
| Oxalic acid | medium | 1125.8 | 2.6 ± 0.1 | 2.9 ± 0.1^*^ | 1.12 | 2.5 ± 0.3 | 3.0 ± 0.3^*^ | 1.23 |
| Urea | medium | 1221.3 | 65.5 ± 24.2 | 78.2 ± 20.7^ns^ | 1.19 | 88.2 ±19.7 | 114.3 ± 17.1^*^ | 1.30 |
| 4-hydroxybutyric acid | medium | 1234.8 | 2.8 ± 0.3 | 5.3 ± 1.1^*^ | 1.85 | 3.3 ± 0.2 | 5.5 ± 0.7^*^ | 1.68 |
| Niacin | medium | 1269.8 | 0.4 ± 0.0 | 0.5 ± 0.0^*^ | 1.21 | 0.5 ± 0.0 | 0.6 ± 0.0^*^ | 1.34 |
| Glycerol | medium | 1292.5 | 26.7 ± 2.1 | 32.9 ± 0.9^*^ | 1.23 | 24.6 ± 3.3 | 31.6 ± 1.4^*^ | 1.28 |
| Threonine | medium | 1294.7 | 49.8 ± 12.7 | 53.5 ± 5.2 ^ns^ | 1.07 | 61.3 ± 10.6 | 80.2 ± 12.6^*^ | 1.31 |
| Succinic acid | medium | 1305.9 | 3.2 ± 0.5 | 3.5 ± 0.1 ^ns^ | 1.09 | 3.4 ± 0.3 | 4.1 ± 0.3^*^ | 1.20 |
| Glycine | medium | 1315.8 | 80.8 ± 20.9 | 102.1 ± 29.4 ^ns^ | 1.26 | 181.4 ± 58.3 | 284.6 ± 47.6^*^ | 1.57 |
| Uracil | medium | 1328.4 | 2.9 ± 0.2 | 4.1 ± 0.1^*^ | 1.41 | 2.9 ± 0.3 | 4.3 ± 0.2^*^ | 1.49 |
| Itaconic acid | medium | 1334.9 | 1.9 ± 0.3 | 6.4 ± 0.6^*^ | 3.25 | 1.6 ± 0.2 | 5.6 ± 0.3^*^ | 3.43 |
| Serine | medium | 1373.9 | 11.9 ± 3.1 | 5.2 ± 1.0^*^ | 0.44 | 15.0 ± 0.4 | 5.4 ± 0.8^*^ | 0.36 |
| Aspartic acid | medium | 1482.2 | 0.3 ± 0.1 | 0.5 ± 0.0 ^ns^ | 1.67 | 0.5 ± 0.1 | 0.8 ± 0.2^*^ | 1.76 |
| Butylated hydroxytoluene | medium | 1492.3 | 2.0 ± 0.0 | 1.3 ± 0.6 ^ns^ | 0.65 | 1.7 ± 0.3 | 1.3 ± 0.1^*^ | 0.77 |
| Malic acid | medium | 1501.2 | 1.3 ± 0.1 | 1.2 ± 0.3 ^ns^ | 0.92 | 1.4 ± 0.1 | 1.6 ±0.1^*^ | 1.17 |
| Pyroglutamic acid | medium | 1506.6 | 1043.4 ± 15.8 | 737.4 ± 43.9^*^ | 0.71 | 1197.2 ± 21.8 | 811.1 ± 12.0^*^ | 0.68 |
| Methionine | medium | 1517.2 | 15.7 ± 4.3 | 12.2 ± 2.5 ^ns^ | 0.77 | 17.1 ± 4.7 | 11.4 ± 0.8^*^ | 0.67 |
| Trihydroxybutyric acid | medium | 1576.4 | 25.9 ± 3.7 | 27.6 ± 0.3 ^ns^ | 1.06 | 24.0 ± 3.0 | 28.0 ± 1.8^*^ | 1.17 |
| Mannose | medium | 1881.4 | 7.0 ± 3.0 | 3.3 ± 0.6 ^*^ | 0.48 | 4.8 ± 2.1 | 2.2 ± 2.0^ns^ | 0.45 |
| Fructose | medium | 1927.7 | 278.2 ± 26.3 | 152.2 ± 20.1^*^ | 0.55 | 273.7 ± 22.6 | 146.2 ± 27.8^*^ | 0.53 |
| Mannitol | medium | 2004.1 | 78.1 ± 6.1 | 79.0 ± 2.7 ^ns^ | 1.01 | 73.0 ± 6.6 | 81.1 ± 5.1^*^ | 1.11 |
| Lactate | lysate | 1059.8 | 0.8 ± 0.1 | 1.3 ± 0.2^*^ | 1.62 | 0.8 ± 0.2 | 1.6 ± 0.2^*^ | 1.89 |
| Oxalic acid | lysate | 1121.3 | 9.0 ± 1.5 | 13.9 ± 3.4^*^ | 1.54 | 10.8 ± 1.5 | 16.4 ± 3.9^*^ | 1.52 |
| Norleucine | lysate | 1149.8 | 92.0 ± 6.9 | 95.7 ± 3.0 ^ns^ | 1.04 | 89.0 ± 2.0 | 101.2 ± 6.1^*^ | 1.14 |
| Hydroxylamine | lysate | 1152.5 | 3.3 ± 0.3 | 3.3 ± 1.1 ^ns^ | 1.01 | 3.6 ± 0.4 | 3.0 ± 0.2^*^ | 0.85 |
| Valine | lysate | 1227.0 | 59.9 ± 17.7 | 70.7 ± 26.0 ^ns^ | 1.18 | 78.0 ± 4.4 | 94.8 ± 8.9^*^ | 1.22 |
| Serine | lysate | 1256.4 | 51.3 ± 4.1 | 26.3 ± 1.3^*^ | 0.51 | 56.6 ± 2.3 | 29.5 ± 1.7^*^ | 0.52 |
| Niacin | lysate | 1272.9 | 1.2 ± 0.3 | 2.5 ± 0.5^*^ | 2.10 | 1.3 ± 0.2 | 2.2 ± 0.4^*^ | 1.77 |
| Glycerol | lysate | 1294.7 | 32.6 ± 9.2 | 32.8 ± 2.3 ^ns^ | 1.01 | 28.9 ± 2.0 | 32.4 ± 1.8^*^ | 1.12 |
| Threonine | lysate | 1297.8 | 133.9 ± 7.0 | 134.0 ± 6.1 ^ns^ | 1.00 | 144.0 ± 3.2 | 158.9 ± 6.7^*^ | 1.10 |
| Uracil | lysate | 1330.7 | 1.5 ± 0.3 | 1.5 ± 0.6 ^ns^ | 1.01 | 1.7 ± 0.2 | 2.1 ± 0.2^*^ | 1.19 |
| Itaconic acid | lysate | 1336.6 | 2.9 ± 0.4 | 9.3 ± 1.1^*^ | 3.25 | 2.6 ± 0.2 | 9.4 ± 0.7^*^ | 3.62 |
| Aspartic acid | lysate | 1415.2 | 91.7 ± 8.6 | 56.3 ± 3.8^*^ | 0.61 | 87.4 ± 5.5 | 47.9 ± 3.5^*^ | 0.55 |
| Malic acid | lysate | 1501.6 | 20.5 ± 1.5 ^ns^ | 21.6 ± 1.3^*^ | 1.05 | 16.6 ± 0.9 | 25.3 ± 1.7^*^ | 1.52 |
| N-acetylglutamate | lysate | 1522.1 | 1282.9 ± 90.8 | 815.2 ± 70.0^*^ | 0.64 | 1068.2 ± 95.0 | 764.1 ± 74.6 ^*^ | 0.72 |
| Threitol | lysate | 1547.9 | 4.3 ± 0.4 | 6.1 ± 0.6^*^ | 1.42 | 4.9 ±0.2 | 7.8 ± 0.2^*^ | 1.60 |
| 2,3,4-Trihydroxybutyric acid | lysate | 1576.4 | 0.3 ± 0.1 | 0.6 ± 0.1^*^ | 2.05 | 0.4 ± 0.0 | 0.7 ± 0.0^*^ | 1.89 |
| 2-Hydroxyglutaric acid | lysate | 1584.4 | 4.9 ± 0.7 | 10.7 ± 1.1^*^ | 2.17 | 4.9 ± 0.6 | 12.2 ± 0.1^*^ | 2.49 |
| Ribitol | lysate | 1796.7 | 3.4 ± 0.5 | 4.1 ± 0.7 ^ns^ | 1.21 | 2.7 ± 0.3 | 3.4 ± 0.3^*^ | 1.29 |
| Citric acid | lysate | 1844.0 | 7.5 ± 0.9 | 8.4 ± 3.3^*^ | 1.12 | 16.6 ± 1.5 | 29.6 ± 2.4^*^ | 1.99 |
| Myristic acid | lysate | 1844.7 | 11.5 ± 1.4 | 13.6 ± 0.8^*^ | 1.19 | 13.9 ± 1.2 | 16.6 ± 1.2^*^ | 1.19 |
| Fructose | lysate | 1928.1 | 16.0 ± 2.4 | 22.0 ± 2.7^*^ | 1.37 | 23.4 ± 2.1 | 31.6 ± 1.3^*^ | 1.35 |
| Myo-Inositol | lysate | 2155.8 | 22.1 ± 2.0 | 34.6 ± 3.4^*^ | 1.57 | 25.5 ± 2.0 | 41.5 ± 2.3^*^ | 1.63 |
| Arachidonic acid | lysate | 2352.5 | 3.0 ± 0.3 | 3.5 ± 0.4^*^ | 1.17 | 3.2 ± 0.1 | 2.7 ± 0.3^*^ | 0.85 |
| 11-Eicosenoic acid | lysate | 2407.5 | 2.1 ± 0.2 | 2.2 ± 0.4 ^ns^ | 1.04 | 2.3 ± 0.1 | 2.1 ± 0.1^*^ | 0.90 |
| Piceatannol | lysate | 2521.1 | 0.7 ± 0.1 | 2.2 ± 0.4^*^ | 3.14 | 0.5 ± 0.1 | 1.7 ± 0.2^*^ | 3.44 |
| Docosahexaenoic acid | lysate | 2552.3 | 3.2 ± 1.4 | 2.5 ± 0.7^*^ | 0.78 | 3.5 ± 0.2 | 4.5 ± 0.3^*^ | 1.29 |
| 2-Monopalmitin | lysate | 2575.8 | 22.8 ± 1.4 | 19.5 ± 2.2^*^ | 0.85 | 26.3 ± 0.7 | 20.5 ± 0.6^*^ | 0.78 |
| 1-Monopalmitin | lysate | 2599.4 | 48.5 ± 3.5 | 42.3 ± 4.3^*^ | 0.87 | 53.5 ± 3.5 | 41.9 ± 1.4^*^ | 0.78 |
| 1-Monooleoylglycerol | lysate | 2765.2 | 0.2 ± 0.1 | 0.3 ± 0.0^*^ | 1.39 | 0.2 ± 0.0 | 0.2 ± 0.0^*^ | 1.27 |
| Stearic acid | lysate | 2792.5 | 72.7 ± 4.4 | 62.4 ± 4.8^*^ | 0.86 | 77.3 ± 6.6 | 59.5 ± 3.8^*^ | 0.77 |
| Uridine monophosphate | lysate | 2836.0 | 0.3 ± 0.1 | 0.8 ± 0.3^*^ | 2.71 | 0.4 ± 0.1 | 0.7 ± 0.2^*^ | 1.63 |
| Cholesta-3,5-diene | lysate | 2885.6 | 3.0 ± 0.3 | 3.1 ± 0.4 ^ns^ | 1.05 | 3.0 ± 0.4 | 1.5 ± 0.5^*^ | 0.52 |
| Eicosanoic acid | lysate | 2983.4 | 2.3 ± 0.2 | 1.9 ± 0.3^*^ | 0.83 | 2.7 ± 0.3 | 2.0 ± 0.1^*^ | 0.73 |
| Zymosterol | lysate | 3202.0 | 4.3 ± 0.4 | 7.6 ± 0.9^*^ | 1.77 | 4.1 ± 0.3 | 6.6 ± 0.7^*^ | 1.59 |

^a^ Metabolite identification using NIST library search.

^b^ Normalized peak area values expressed as mean ± S.E.M.

^c^ Fold change (Δ): CHO-AβPP_695 (treatment)_ **/** CHO-wt _(treatment)_.

* *p*<0.05 and ^ns^ not significant when calculated using the independent *t*-test with Welch’s correction for normalized peak area of CHO-AβPP_695_ cells compared to CHO-wt cells for respective treatment groups.
