# Supplementary material for: Metabotyping of Docosahexaenoic Acid - Treated Alzheimer’s Disease Cell Model
Source: PLoS One. 2014 Feb 27;9(2):e90123. doi: 10.1371/journal.pone.0090123 (PMC3937442; doi:10.1371/journal.pone.0090123)
Supplement: Text S1 — Methods for Sample Preparation and Derivatization. (DOCX) [file pone.0090123.s004.docx]

**Supporting information - Text S1: Methods for Sample Preparation and Derivatization**

Prior to derivatization, all the samples were thawed at room temperature (24 ± 1°C). For culture medium, 200 µL of each sample was mixed with 150 µL of 100 µM mefenamic acid in water (internal standard) and 1 mL methanol and vortex-mixed at a high speed for 5 min. All the tubes were subsequently centrifuged at 10,000*g* for 10 min at 4°C and 950 µL of supernatant from each sample was transferred into 15 mL silylated glass tubes. Likewise, for cell lysate, 300 µL of 100 µM mefenamic acid in water and 200 µL of methanol were added to the pooled cells in 1 mL cold methanol and cell disruption was performed with a ball mill for 5 min at 25 Hz using 4 mm stainless steel beads. The extracts were then placed on ice for 20 min, followed by centrifugation at 17,500*g* for 10 min at 4°C. 1.4 mL of each supernatant was transferred to silanized glass tubes. The collected supernatants were then evaporated to complete dryness followed by methoximation and trimethylsilyl derivatization.
